# *Toxoplasma gondii*‐Derived Soluble Factors Induce G2/M Phase Accumulation and p53‐Associated Mitochondrial Apoptosis in Human Umbilical Cord Mesenchymal Stem Cells

**DOI:** 10.1111/jcmm.71312

**Published:** 2026-08-16

**Authors:** Wei Zhou, Tingyu Chen, Ziping Yang, Jinjing Huang, Xinyi Lai, Jie Liu, Jiaqi Chu, Juanhua Quan

**Affiliations:** ^1^ Biotissue Repository Affiliated Hospital of Guangdong Medical University Zhanjiang Guangdong China; ^2^ Department of Gastroenterology Affiliated Hospital of Guangdong Medical University Zhanjiang Guangdong China; ^3^ Experimental Animal Center Affiliated Hospital of Guangdong Medical University Zhanjiang Guangdong China; ^4^ Stem Cell Research and Cellular Therapy Center Affiliated Hospital of Guangdong Medical University Zhanjiang Guangdong China; ^5^ Laboratory of Gastroenterology Affiliated Hospital of Guangdong Medical University Zhanjiang Guangdong China

**Keywords:** G2/M phase accumulation, hUC‐MSCs, mitochondrial apoptosis, parasite‐derived soluble factors, *Toxoplasma gondii*

## Abstract

*Toxoplasma gondii* infection poses potential risks to stem cell‐based therapeutic applications; however, the effects of parasite‐derived soluble factors on mesenchymal stem cells remain unclear. Here, we investigated the impact of *T. gondii*‐derived soluble factors (Tg‐SFs) on human umbilical cord‐derived mesenchymal stem cells (hUC‐MSCs). hUC‐MSCs were exposed to Tg‐SFs generated in a Transwell co‐culture system, and cellular phenotypes and p53 signalling were analysed using integrated functional and molecular approaches. Tg‐SFs induced upper‐chamber multiplicity of infection (MOI)‐dependent cytotoxicity in hUC‐MSCs, accompanied by cytoskeletal remodelling and nuclear condensation. Tg‐SFs exposure caused marked G2/M phase accumulation, which was accompanied by reduced expression of Cyclin A2, Cyclin B1, and Cyclin E2 and increased p21 expression. In addition, Tg‐SFs exposure reduced phosphorylation of CDK1 at Thr161. Tg‐SFs‐exposed hUC‐MSCs underwent significant apoptosis, as evidenced by PARP and Caspase‐3 cleavage. Mechanistically, Tg‐SFs promoted accumulation of total p53 protein and selective phosphorylation of p53 at Ser15 and Ser392, and modulated Bcl‐2 family proteins by upregulating Bax, BID, Bak, Bad, and Puma while downregulating Bcl‐xL and Mcl‐1. Pharmacological inhibition of p53 with PFT‐α and siRNA‐mediated p53 knockdown partially reversed these effects, indicating that p53 signalling contributes substantially, but not exclusively, to Tg‐SFs‐induced mitochondrial apoptosis. These findings indicate that *T. gondii*‐derived soluble factors impair hUC‐MSC viability through G2/M phase accumulation and p53‐associated mitochondrial apoptosis, highlighting a potential safety consideration for MSC‐based applications under parasite‐associated conditions.

## 1 Introduction

*Toxoplasma gondii* is an obligate intracellular protozoan parasite capable of infecting nearly all warm‐blooded animals. Approximately one‐third of the global population is seropositive, and infection remains a major public health concern due to lifelong persistence and association with ocular disease, congenital toxoplasmosis, and severe opportunistic infections in immunocompromised individuals [1]. These clinical manifestations reflect the parasite's capacity to establish latent infection and modulate host cellular homeostasis.

Accumulating evidence indicates that *T. gondii* infection interferes with fundamental cellular processes, including cytoskeletal organisation, cell‐cycle regulation, and stress‐response signalling, which may impact tissue regeneration and stem‐cell therapies [2]. A defining feature of *T. gondii* biology is secretion of excretory/secretory products (ESPs), a complex mixture of dense granule proteins, secreted effectors, and vesicle‐like components released during motility, host attachment, invasion, and intracellular development [3, 4]. ESPs function as autonomous signalling entities that can exert immunomodulatory and cytotoxic effects even in the absence of intracellular parasitism [5].

Previous studies have demonstrated that *T. gondii* infection disrupts host cytoskeletal organization and cell‐cycle progression, leading to altered proliferation and arrest [6, 7]. Recent studies suggested that parasite‐derived soluble factors and extracellular vesicles (EVs) can induce abnormalities in neighbouring, non‐infected host cells [8]. However, it remains unclear whether parasite‐derived soluble factors released from *T. gondii* tachyzoites, independent of direct parasite invasion, are sufficient to compromise mesenchymal stem cells (MSCs) homeostasis or regulate survival pathways in MSCs [9, 10].

MSCs, particularly those derived from human umbilical cord (hUC‐MSCs), are widely utilized in regenerative medicine owing to their low immunogenicity, proliferative capacity, multilineage potential, and potent immunomodulatory effects [11, 12, 13]. MSCs are sensitive to environmental stressors, including pathogen‐associated molecular patterns and microbial products, which can affect viability and function. Our previous work revealed that direct *T. gondii* infection triggers autophagy and apoptosis in hUC‐MSCs through down‐regulation of the anti‐apoptotic Bcl‐2 family protein Mcl‐1, indicating that active infection disrupts MSCs' homeostasis [14]. However, whether *T. gondii* ESPs alone perturb MSC survival and regulatory pathways remains unexplored.

G2/M arrest is a critical stress response preventing mitotic entry and can trigger apoptosis if damage is irreparable [15]. The tumour suppressor p53 regulates the G2/M checkpoint and mitochondrial apoptosis by controlling p21 and Bcl‐2 family protein expression [16]. Although p53 activation has been reported following *T. gondii* infection, its role in ESP‐mediated cytotoxicity in MSCs remains largely unexplored [17].

Given the translational relevance of MSCs and the global prevalence of latent or active *T. gondii* infection, we aimed to investigate the effects of parasite‐derived soluble factors (Tg‐SFs) on hUC‐MSC viability, cytoskeletal integrity, cell‐cycle progression, and apoptosis using a Transwell system. We also sought to elucidate the underlying molecular mechanisms, with a particular focus on p53‐associated mitochondrial apoptotic signalling. Our findings indicate that parasite‐derived soluble factors released from *T. gondii* tachyzoites are sufficient to induce G2/M phase accumulation, cytoskeletal remodelling, and mitochondrial apoptosis, with p53 signalling contributing substantially, but not exclusively, to these effects. These findings reveal a previously unrecognised invasion‐independent mechanism by which parasite‐derived soluble factors alter MSC homeostasis and highlight potential safety considerations for MSC‐based therapeutic applications.

## 2 Materials and Methods

### 2.1 Isolation, Culture and Characterisation of hUC‐MSCs

Human umbilical cord‐derived mesenchymal stem cells (hUC‐MSCs) were isolated and cultured as previously described [14]. Cells were maintained in low‐glucose DMEM supplemented with 10% FBS at 37°C in a humidified atmosphere containing 5% CO_2_. Non‐adherent cells were removed after 48 h, and the medium was replaced every 3 days. Phenotypic characterisation was performed using a Human MSC Analysis Kit (BD Biosciences) and analysed by flow cytometry.

### 2.2 Maintenance of *T. gondii*

*T. gondii* RH strain tachyzoites were maintained as previously described with minor modifications [18]. Parasites were harvested following host cell lysis, purified by needle passage, and filtered through a 5.0‐μm membrane to remove host cell debris.

### 2.3 Transwell Co‐Culture System

A Transwell co‐culture system (0.4‐μm pore size) was established as previously described [19]. hUC‐MSCs were seeded in the lower chamber, whereas live *T. gondii* RH strain tachyzoites were added to the upper chamber at the indicated upper‐chamber MOIs and co‐cultured for 24 h. The 0.4‐μm membrane prevented direct parasite invasion while allowing hUC‐MSCs to be exposed to parasite‐derived soluble factors (Tg‐SFs).

For these experiments, 0.4‐μm PET membrane inserts (Corning, Cat. No. 3450 for 6‐well plates and Cat. No. 3460 for 12‐well plates) were used for siRNA transfection, Western blot analysis, or immunofluorescence staining, respectively.

### 2.4 Cell Viability and Morphological Analysis

Cell viability was assessed using the CellTiter 96 AQueous One Solution Cell Proliferation Assay Kit according to the manufacturer's instructions. For morphological analysis, hUC‐MSCs grown on coverslips were fixed, permeabilized, and stained with Texas Red‐X phalloidin to visualize F‐actin, followed by DAPI nuclear counterstaining.

### 2.5 Preparation of *T. gondii* Excretory/Secretory Products (ESPs)

ESPs were prepared as previously described with minor modifications [20]. Purified *T. gondii* tachyzoites were incubated in Hank's balanced salt solution for 3 h. Supernatants were collected by centrifugation, aliquoted, and stored at −80°C. Protein concentrations were determined using a BCA protein assay kit. Equal amounts of protein (30 μg per lane) from Lys and ESP preparations were used for SDS‐PAGE and Western blot analyses unless otherwise indicated.

### 2.6 SDS‐PAGE and Western Blotting Analysis

ESP proteins were resolved by SDS‐PAGE and visualized by Coomassie Brilliant Blue G‐250 staining. For Western blotting, total cellular proteins were extracted, separated by SDS‐PAGE, and transferred to PVDF membranes. Membranes were probed with antibodies against cell cycle regulators, total p53, phosphorylated p53, apoptosis‐related proteins, and GAPDH. Protein signals were detected by chemiluminescence and quantified by densitometric analysis using ImageJ software after normalisation to the corresponding loading controls or total protein levels, as appropriate.

### 2.7 Transmission Electron Microscopy

ESP samples were fixed, embedded, ultrathin‐sectioned, and examined using a JEM‐1400 transmission electron microscope according to standard protocols.

### 2.8 Immunofluorescence and Confocal Microscopy

hUC‐MSCs were exposed to *T. gondii*‐derived soluble factors (Tg‐SFs), defined as parasite‐derived soluble factors released from live *T. gondii* tachyzoites placed in the upper chamber of the Transwell co‐culture system (upper‐chamber MOI 50), for 24 h. For TP3 staining, direct infection with live RH tachyzoites (MOI 5) was included as a positive control. Cells were fixed and incubated with primary antibodies against TP3, α‐Tubulin, or p53 as indicated. Fluorescent images were captured using confocal microscopy.

### 2.9 Quantitative Real‐Time PCR

Total RNA was extracted, reverse‐transcribed, and subjected to quantitative real‐time PCR using Power SYBR Green PCR Master Mix. Relative gene expression levels were calculated using the 2^−ΔΔCt^ method with HPRT1 as the reference gene. Primer sequences are listed in Table 1.

**TABLE 1:** Primer sequences used for quantitative real‐time PCR.

Gene	Primer sequence (5′‐3′)	Product size (bp)
Cyclin A2	Forward: CTCTACACAGTCACGGGACAAAG	120
	Reverse: CTGTGGTGCTTTGAGGTAGGTC	
Cyclin B1	Forward: GACCTGTGTCAGGCTTTCTCTG	154
	Reverse: GGTATTTTGGTCTGACTGCTTGC	
Cyclin E2	Forward: CTTACGTCACTGATGGTGCTTGC	126
	Reverse: CTTGGAGAAAGAGATTTAGCCAGG	
Myt1	Forward: TTCAGACCAGCGAAACCTCACC	145
	Reverse: TGTGGCTTCGTGCTGAGGTTCT	
HPRT1	Forward: CATTATGCTGAGGATTTGGAAAGG	129
	Reverse: CTTGAGCACACAGAGGGCTACA	

### 2.10 Cell Cycle and Apoptosis Analysis

Cell cycle distribution was analysed by propidium iodide staining. Apoptosis was evaluated using the FITC Annexin V Apoptosis Detection Kit, followed by flow cytometric analysis. Flow cytometry data were analysed using FlowJo software. The gating strategy for Annexin V‐FITC/PI apoptosis analysis is shown in Figure S1.

### 2.11 Assessment of Mitochondrial Membrane Potential

Mitochondrial membrane potential was assessed using the JC‐1 MitoMP detection kit and analysed by confocal microscopy.

### 2.12 siRNA Transfection

hUC‐MSCs were cultured in 6‐well plates to 60%–70% confluence at 37°C in a humidified atmosphere containing 5% CO₂. Cells were transfected with control siRNA (sc‐37,007) or p53 siRNA (sc‐29,435) using siRNA Transfection Reagent (sc‐29,528) and siRNA Transfection Medium (sc‐36,868; Santa Cruz Biotechnology, Dallas, TX, USA) according to the manufacturer's instructions. Briefly, 60 pmol siRNA and 6 μL transfection reagent were diluted separately, mixed, and incubated for 20 min at room temperature to allow formation of transfection complexes. After 6 h of incubation, an equal volume of 2× complete medium was added without removing the transfection complexes. Forty‐eight hours after transfection, cells were exposed to *T. gondii*‐derived soluble factors (Tg‐SFs), defined as parasite‐derived soluble factors released from live *T. gondii* tachyzoites in the upper chamber of the Transwell co‐culture system (upper‐chamber MOI 50), or maintained under control conditions for 24 h before being harvested for Western blot analysis.

### 2.13 Statistical Analysis

Data are presented as mean ± standard deviation (SD) from three independent biological experiments (*n* = 3 biological replicates) unless otherwise indicated. Statistical analyses were performed using GraphPad Prism 10.0 software (GraphPad Software, San Diego, CA, USA). Comparisons between two groups were performed using an unpaired two‐tailed Student's *t*‐test, whereas comparisons among multiple groups were analysed by one‐way ANOVA with appropriate post hoc tests. For fluorescence image analysis, fluorescence intensity was quantified on a per‐cell basis using ImageJ software. A value of *p* < 0.05 was considered statistically significant.

Additional experimental details regarding qRT‐PCR, Western blotting, flow cytometry, statistical analysis, and endotoxin detection are provided in the Data S1.

## 3 Results

### 3.1 *T. gondii*‐Derived Soluble Factors Induce Cytotoxicity and Cytoskeletal Remodelling in hUC‐MSCs

**FIGURE 1:**
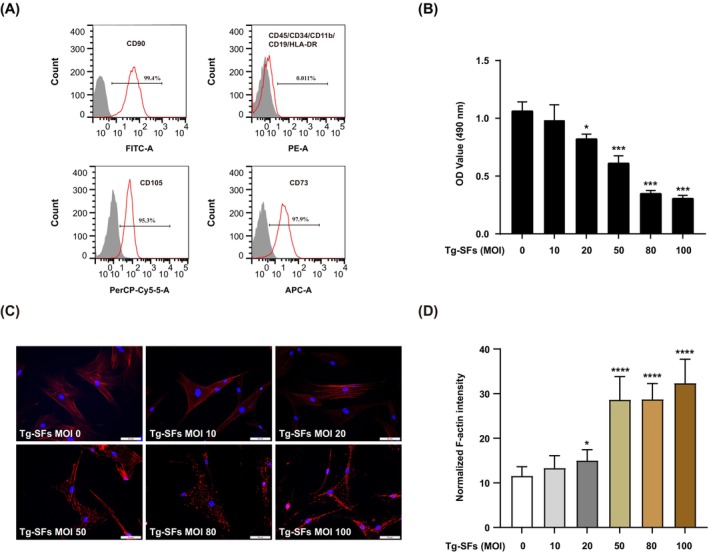
*Toxoplasma gondii*‐derived soluble factors induce cytotoxicity and cytoskeletal remodelling in hUC‐MSCs. (A) Flow cytometric characterisation of hUC‐MSCs showing high expression of mesenchymal stem cell (MSC) markers CD90, CD105, and CD73 (> 95%) and absence of haematopoietic lineage markers CD45, CD34, CD11b, CD19, and HLA‐DR. (B) Cell viability of hUC‐MSCs following 24 h exposure to *T. gondii*‐derived soluble factors (Tg‐SFs), defined as parasite‐derived soluble factors released from live *T. gondii* tachyzoites placed in the upper chamber of the Transwell co‐culture system, generated at upper‐chamber multiplicities of infection (MOIs) of 0, 10, 20, 50, 80, or 100, assessed using an MTS assay. Data are presented as mean ± SD. *p < 0.05, ***p < 0.001 versus the control group. (C) Representative immunofluorescence analysis of F‐actin (Texas Red‐phalloidin, red) and nuclei (DAPI, blue) in hUC‐MSCs exposed to Tg‐SFs. Tg‐SFs induced marked cytoskeletal remodelling characterized by altered F‐actin organization increased actin aggregation, and changes in cellular morphology. Scale bar = 50 μm. (D) Quantitative analysis of F‐actin fluorescence intensity per cell. Fluorescence intensity was calculated as integrated density/area from the F‐actin fluorescence channel using ImageJ. At least 20 individual cells were analysed per group following exposure to Tg‐SFs generated at upper‐chamber MOIs of 0, 10, 20, 50, 80, and 100. Data are presented as mean ± SD. Bartlett's test confirmed unequal variances across groups, so statistical significance was determined by Brown‐Forsythe corrected one‐way ANOVA followed by Dunnett's post hoc test for comparisons versus the MOI = 0 control group. *p < 0.05, ****p < 0.0001 versus the control group. Tg‐SFs: Parasite‐derived soluble factors released from live *T. gondii* tachyzoites placed in the upper chamber of the Transwell co‐culture system.

Human umbilical cord‐derived mesenchymal stem cells (hUC‐MSCs) were isolated by adherence separation and characterised by flow cytometry prior to experimental use. The cells exhibited typical fibroblast‐like morphology and robust expression of MSC surface markers CD90, CD105, and CD73 (> 95%), while lacking expression of haematopoietic markers CD45, CD34, CD11b, CD19, and HLA‐DR (Figure 1A), confirming their mesenchymal identity. Given our previous observation that direct *T. gondii* infection compromises hUC‐MSC viability through autophagy‐associated apoptosis [14], we next investigated whether parasite‐derived soluble factors could exert cytotoxic effects independently of direct parasite invasion.

Using a Transwell co‐culture system, hUC‐MSCs were exposed for 24 h to *T. gondii*‐derived soluble factors (Tg‐SFs), defined as parasite‐derived soluble factors released from live *T. gondii* tachyzoites placed in the upper chamber, at upper‐chamber multiplicities of infection (MOIs) of 10–100. Cell viability was significantly reduced in an upper‐chamber MOI‐dependent manner, with significant decreases observed at upper‐chamber MOIs of 20 and above and the strongest suppression detected at MOIs of 80–100 (Figure 1B).

Morphological analysis revealed that control hUC‐MSCs displayed organised F‐actin stress fibres and normal nuclear morphology. In contrast, Tg‐SFs exposure induced progressive cytoskeletal remodelling, characterized by redistribution of F‐actin, increased actin aggregation, loss of organized stress fibre architecture, marked alterations in cell morphology at higher upper‐chamber MOIs (Figure 1C). Quantitative image analysis further demonstrated a dose‐dependent increase in F‐actin fluorescence intensity per cell following Tg‐SFs exposure, with significant elevations observed at upper‐chamber MOIs ≥ 20 and pronounced increases at MOIs of 50–100, supporting the cytoskeletal remodelling and actin aggregation observed by immunofluorescence microscopy (Figure 1D). Collectively, these findings indicate that parasite‐derived soluble factors released from live *T. gondii* tachyzoites are sufficient to induce cytotoxicity and cytoskeletal alterations in hUC‐MSCs in the absence of direct parasite invasion.

### 3.2 Biochemical and Ultrastructural Characterisation of In Vitro‐Prepared *T. gondii* Excretory‐Secretory Products (ESPs)

**FIGURE 2:**
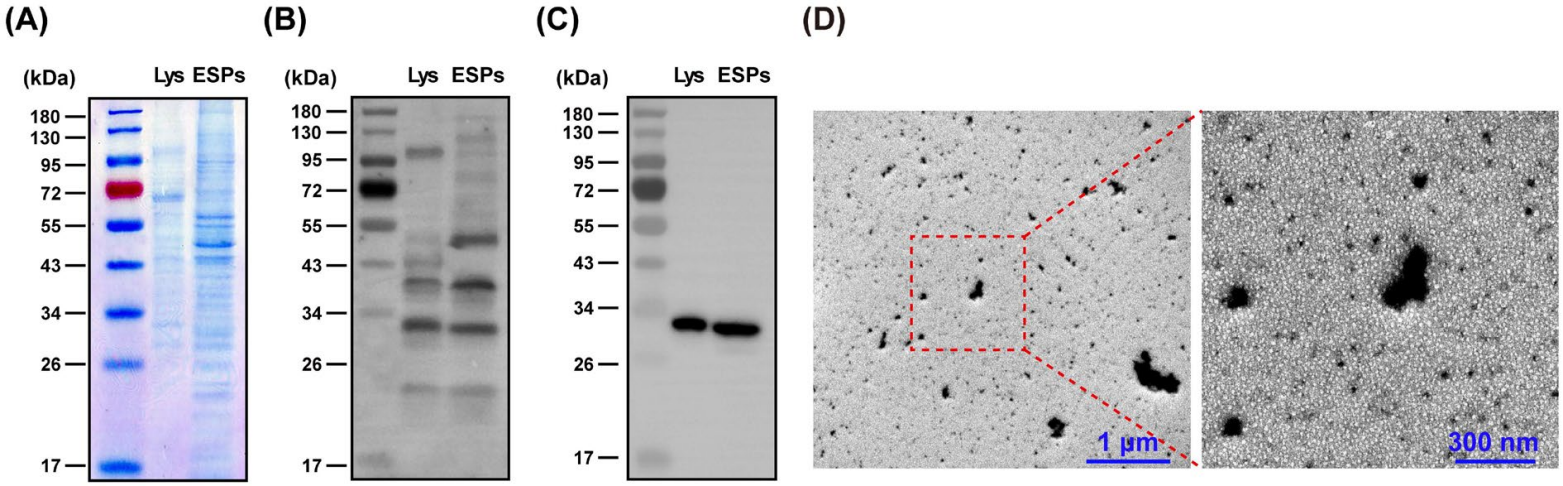
Biochemical and ultrastructural characterisation of in vitro‐prepared *Toxoplasma gondii* excretory‐secretory products (ESPs). Equal amounts of protein (30 μg per lane) from Lys and ESP preparations were loaded for SDS‐PAGE and Western blot analyses. (A) Coomassie Brilliant Blue G‐250 staining of SDS‐PAGE‐separated proteins showing the overall protein profiles of *T. gondii* tachyzoite lysates (Lys) and in vitro‐prepared ESPs. (B) Western blotting analysis using *T. gondii*‐positive human sera demonstrating multiple immunoreactive antigens in both Lys and ESP preparations. (C) Western blotting analysis using a commercially available *T. gondii*‐specific antibody (TP3) confirming the presence of parasite‐derived proteins in Lys and ESP preparations. (D) Ultrastructural morphology of in vitro‐prepared ESPs examined by transmission electron microscopy.

ESPs were prepared in vitro, with tachyzoite lysates used as a positive control. Coomassie Brilliant Blue staining revealed a complex protein profile in the ESP fraction, exhibiting greater diversity than whole‐parasite lysates (Figure 2A). Western blotting using *T. gondii*‐positive human sera identified multiple immunoreactive bands in both preparations (Figure 2B), confirming the presence of antigens naturally recognised during human infection. In addition, the *T. gondii*‐specific antibody TP3 detected a prominent protein band in both ESPs and lysates (Figure 2C). Transmission electron microscopy revealed that the ESP preparation contained abundant nanoscale vesicle‐like structures with intact membrane boundaries, along with heterogeneous electron‐dense materials (Figure 2D). Collectively, these results confirm the complexity, antigenicity, and preserved ultrastructure of the in vitro‐prepared *T. gondii* ESPs, demonstrating the successful preparation of ESP samples for biochemical and ultrastructural characterisation.

### 3.3 *T. gondii*‐Derived Soluble Factors Induce G2/M Phase Accumulation and Apoptosis in hUC‐MSCs

**FIGURE 3:**
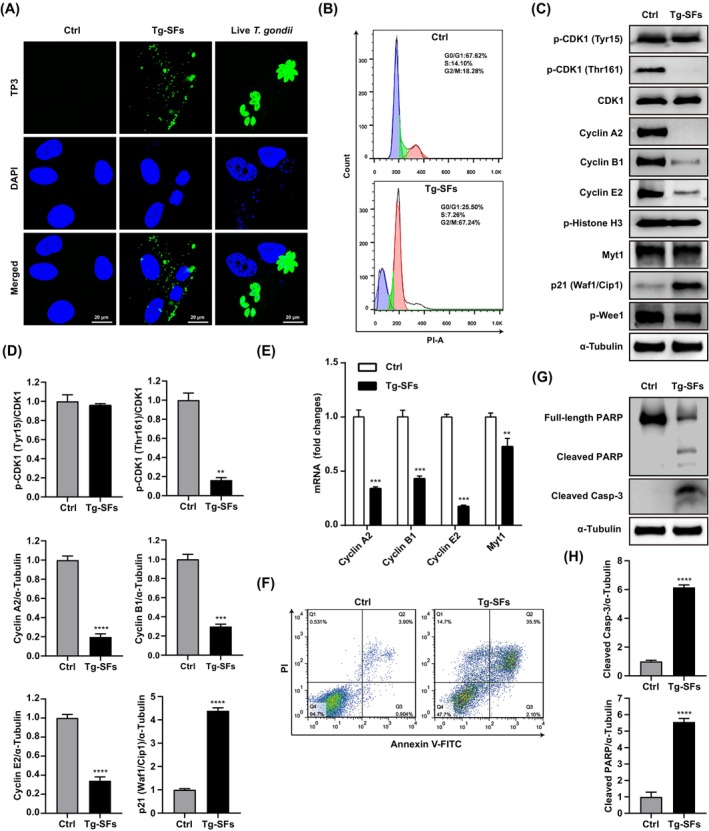
*Toxoplasma gondii*‐derived soluble factors induce G2/M accumulation and apoptosis in hUC‐MSCs. (A) hUC‐MSCs were exposed to parasite‐derived soluble factors released from live *T. gondii* tachyzoites in the upper chamber of the Transwell co‐culture system (upper‐chamber MOI 50) for 24 h or directly infected with live RH tachyzoites (MOI 5, positive control). Parasite‐derived antigens were detected by TP3 immunostaining and visualised by confocal microscopy (green). (B) Cell cycle distribution of hUC‐MSCs was analysed by propidium iodide (PI) staining followed by flow cytometry. Percentages of G0/G1, S, and G2/M phase populations are shown. (C) Protein expression levels of cell cycle‐related regulators, including p‐CDK1 (Tyr15), p‐CDK1 (Thr161), CDK1, Cyclin A2, Cyclin B1, Cyclin E2, p‐Histone H3, Myt1, p21 (Waf1/Cip1), and p‐Wee1, were assessed by Western blotting. (D) Quantitative densitometric analysis of p‐CDK1 (Thr161)/CDK1, Cyclin A2, Cyclin B1, Cyclin E2, and p21 (Waf1/Cip1) protein expression shown in panel (C). Data are presented as mean ± SD. **p < 0.01, ***p < 0.001, ****p < 0.0001 versus the control group. (E) Relative mRNA expression levels of Cyclin A2, Cyclin B1, Cyclin E2, and Myt1 were determined by qRT‐PCR. Data are presented as the mean ± SD from three independent experiments. **p < 0.01, ***p < 0.001 versus the control group. (F) Apoptotic cell populations were quantified by Annexin V‐FITC/PI staining followed by flow cytometry. (G) Expression levels of PARP and cleaved caspase‐3 were analysed by Western blotting. (H) Quantitative densitometric analysis of cleaved PARP and cleaved caspase‐3 expression shown in panel (G). Protein levels were normalized to α‐Tubulin and expressed relative to the control group. Data are presented as mean ± SD. ****p < 0.0001 versus the control group. Representative Western blot images from one of three independent biological experiments are shown. Densitometric quantification was performed using data from all three independent biological experiments. Tg‐SFs: Parasite‐derived soluble factors released from live *T. gondii* tachyzoites placed in the upper chamber of the Transwell co‐culture system.

To confirm cellular exposure to parasite‐derived components, hUC‐MSCs were exposed to *T. gondii*‐derived soluble factors (Tg‐SFs), defined as parasite‐derived soluble factors released from live *T. gondii* tachyzoites placed in the upper chamber of the Transwell co‐culture system (at an upper‐chamber MOI of 50) for 24 h. TP3 immunofluorescence staining revealed abundant punctate TP3‐positive signals in Tg‐SFs‐treated cells, whereas direct infection with live tachyzoites produced the characteristic intracellular crescent‐shaped staining pattern (Figure 3A), confirming exposure of hUC‐MSCs to parasite‐derived materials in the absence of direct parasite invasion.

Flow cytometric analysis demonstrated that Tg‐SFs exposure caused a pronounced G2/M phase accumulation. The proportion of cells in G2/M phase increased from 18.1% ± 0.2% in control cells to 66.7% ± 4.6% following Tg‐SFs exposure, accompanied by reductions in G0/G1 and S phase populations (Figure 3B). Consistent with these findings, Western blotting revealed decreased expression of Cyclin A2, Cyclin B1, and Cyclin E2, along with marked upregulation of the cyclin‐dependent kinase inhibitor p21 (Waf1/Cip1). In addition, Tg‐SFs exposure resulted in reduced phosphorylation of CDK1 at Thr161, whereas total CDK1 and p‐CDK1 (Tyr15) levels remained largely unchanged (Figure 3C,D). These findings are consistent with impaired CDK1 activation associated with G2/M phase accumulation. Quantitative qRT‐PCR analysis further demonstrated significant downregulation of Cyclin A2, Cyclin B1, Cyclin E2, and Myt1 transcripts in Tg‐SFs‐treated cells (Figure 3E).

To determine whether G2/M phase accumulation was associated with cell death, apoptosis was assessed by Annexin V‐FITC/PI staining. Tg‐SFs exposure markedly increased late apoptotic cell populations (34.82% ± 3.1% vs. 2.89% ± 1.0% in control cells; Figure 3F). Consistently, Western blotting demonstrated that Tg‐SFs exposure induced PARP cleavage and robust activation of cleaved caspase‐3 in Tg‐SFs‐treated cells (Figure 3G,H), confirming induction of apoptotic signalling. Collectively, these results indicate that parasite‐derived soluble factors released from live *T. gondii* tachyzoites induce G2/M phase accumulation and apoptosis in hUC‐MSCs, accompanied by dysregulation of key cell‐cycle regulators and activation of the apoptotic machinery.

### 3.4 *T. gondii*‐Derived Soluble Factors Activate p53 Signalling and Alter Bcl‐2 Family Protein Expression

**FIGURE 4:**
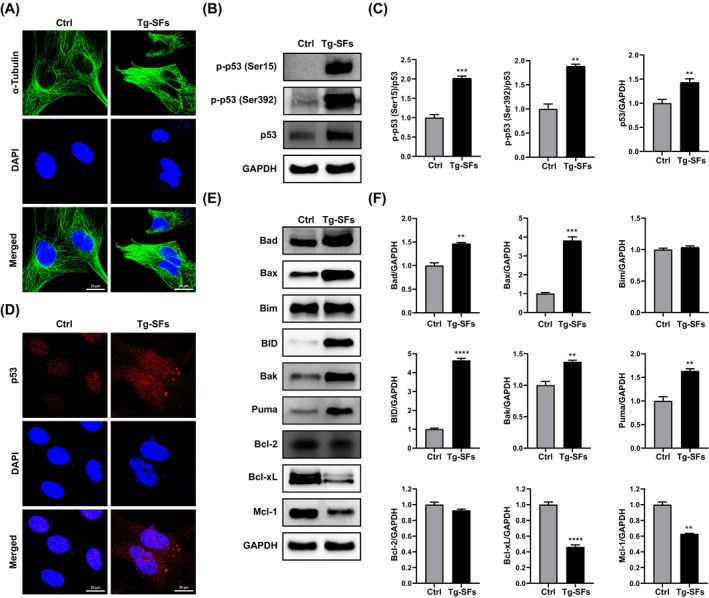
*Toxoplasma gondii*‐derived soluble factors activate p53 signalling and alter Bcl‐2 family protein expression in hUC‐MSCs. hUC‐MSCs were exposed to Tg‐SFs, defined as parasite‐derived soluble factors released from live *T. gondii* tachyzoites placed in the upper chamber of the Transwell co‐culture system at an upper‐chamber MOI of 50 for 24 h. (A) Microtubule organisation was visualised by confocal microscopy following α‐Tubulin immunostaining. Representative images show intact filamentous microtubule networks in control cells and disrupted microtubule networks in Tg‐SFs‐treated cells. Scale bar = 20 μm. (B) Western blotting analysis of p‐p53 (Ser15), p‐p53 (Ser392), total p53, and GAPDH in control and Tg‐SFs‐treated hUC‐MSCs. GAPDH served as the loading control. (C) Quantitative densitometric analysis of p‐p53 (Ser15), p‐p53 (Ser392), and total p53 protein expression shown in panel (B). Phosphorylated p53 levels were normalized to total p53, whereas total p53 was normalized to GAPDH. Data are presented as mean ± SD. **p < 0.01, ***p < 0.001, ****p < 0.0001 versus the control group. (D) Immunofluorescence staining of p53 (red) and nuclei (DAPI, blue) showing increased p53 expression and intracellular accumulation in Tg‐SFs‐treated hUC‐MSCs. Scale bar = 20 μm. (E) Western blotting analysis of Bcl‐2 family proteins, including Bad, Bax, Bim, BID, Bak, Puma, Bcl‐2, Bcl‐xL, and Mcl‐1, in control and Tg‐SFs‐treated hUC‐MSCs. GAPDH served as the loading control. (F) Quantitative densitometric analysis of Bcl‐2 family protein expression shown in panel (E). Protein levels were normalized to GAPDH and expressed relative to the control group. Data are presented as mean ± SD. **p < 0.01, ***p < 0.001, ****p < 0.0001 versus the control group. Representative Western blot images from one of three independent biological experiments are shown. Densitometric quantification was performed using data from all three independent biological experiments. Tg‐SFs: Parasite‐derived soluble factors released from live *T. gondii* tachyzoites placed in the upper chamber of the Transwell co‐culture system.

Immunofluorescence staining revealed that Tg‐SFs exposure caused severe disruption of the α‐Tubulin network, indicative of microtubule destabilisation (Figure 4A). Western blotting analysis demonstrated that Tg‐SFs selectively increased p53 phosphorylation at Ser15 and Ser392, accompanied by accumulation of total p53 protein (Figure 4B). Quantitative densitometric analysis confirmed that the levels of p‐p53 (Ser15)/p53, p‐p53 (Ser392)/p53, and total p53/GAPDH were significantly increased in Tg‐SFs‐treated cells compared with control cells (Figure 4C). Additional p53 phosphorylation sites were examined using a phospho‐p53 antibody panel. However, p‐p53 (Ser6), p‐p53 (Ser9), p‐p53 (Ser46), and p‐p53 (Thr81) were not detected at the expected molecular weight, whereas p‐p53 (Ser20) showed no obvious change following Tg‐SFs exposure. These data are presented in Figure S2. Immunofluorescence analysis further confirmed marked upregulation and intracellular accumulation of p53 in Tg‐SFs‐treated hUC‐MSCs (Figure 4D). Analysis of mitochondrial apoptosis regulators showed that Tg‐SFs exposure significantly upregulated the pro‐apoptotic proteins Bad, Bax, BID, Bak, and Puma, while downregulating the anti‐apoptotic proteins Bcl‐xL and Mcl‐1 (Figure 4E). Quantitative densitometric analysis further verified these expression changes, demonstrating significant increases in Bad, Bax, BID, Bak, and Puma protein levels and significant decreases in Bcl‐xL and Mcl‐1 expression following Tg‐SFs exposure (Figure 4F). In contrast, the expression levels of Bim and Bcl‐2 remained largely unchanged (Figure 4E,F). Taken together, these findings demonstrate that Tg‐SFs activate p53 signalling, characterised by increased p53 accumulation and phosphorylation at Ser15 and Ser392, and promote mitochondrial apoptotic signalling through coordinated modulation of Bcl‐2 family proteins, thereby favouring a pro‐apoptotic cellular state in hUC‐MSCs.

### 3.5 p53 Partially Mediates Tg‐SFs‐Induced Mitochondrial Apoptosis in hUC‐MSCs

**FIGURE 5:**
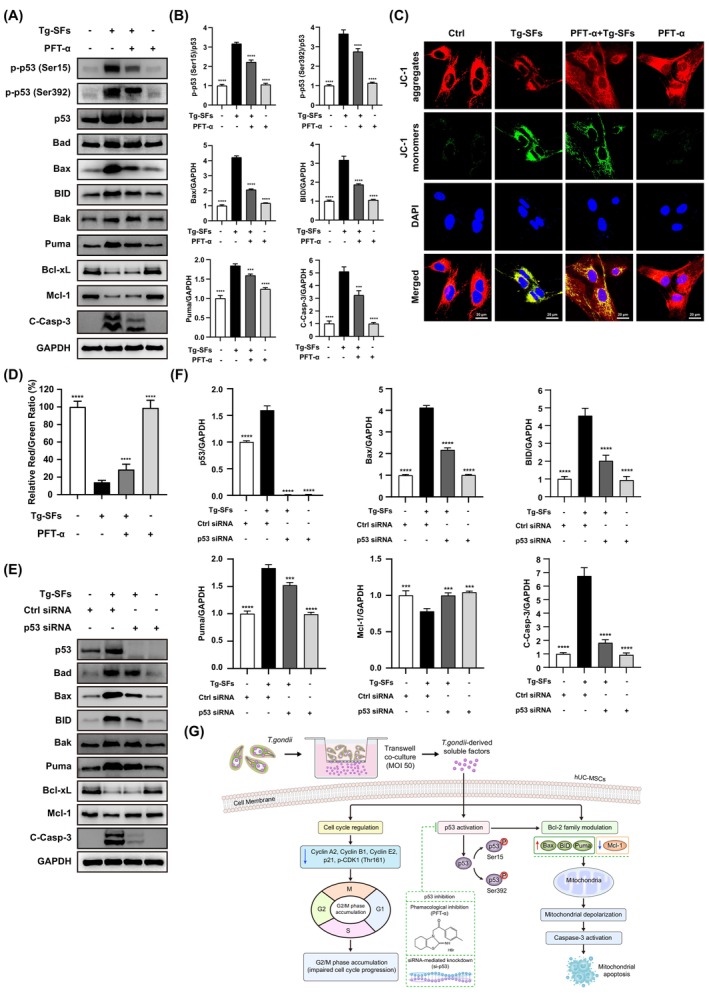
Pharmacological inhibition and siRNA‐mediated knockdown of p53 attenuate Tg‐SFs‐induced mitochondrial apoptosis in hUC‐MSCs. hUC‐MSCs were pretreated with the p53 inhibitor pifithrin‐α (PFT‐α, 10 μM) for 2 h prior to exposure to Tg‐SFs (upper‐chamber MOI 50) for 24 h. For genetic inhibition, hUC‐MSCs were transfected with control siRNA or p53 siRNA before exposure to Tg‐SFs. (A) Representative Western blotting analysis showing phosphorylated p53 (Ser15 and Ser392), Bcl‐2 family proteins (Bad, Bax, BID, Bak, Puma, Bcl‐xL, and Mcl‐1), and cleaved caspase‐3 (C‐Casp‐3). GAPDH served as the loading control. (B) Quantitative densitometric analysis of the selected proteins shown in panel (A), including p‐p53 (Ser15)/p53, p‐p53 (Ser392)/p53, Bax, BID, Puma, and C‐Casp‐3. Phosphorylated p53 levels were normalized to total p53, whereas the remaining proteins were normalized to GAPDH. Data are presented as mean ± SD. ***p < 0.001, ****p < 0.0001 versus the Tg‐SFs group (ordinary one‐way ANOVA followed by Dunnett's multiple comparisons test). (C) Mitochondrial membrane potential (Δψ*m*) was assessed by JC‐1 staining and visualised by confocal microscopy. Cells with preserved Δψ*m* display red JC‐1 aggregates, whereas cells with mitochondrial depolarization exhibit green JC‐1 monomers. (D) Quantitative analysis of mitochondrial membrane potential (Δψ*m*) based on the JC‐1 red/green fluorescence intensity ratio. Data are presented as mean ± SD. ****p < 0.0001 vs. the Tg‐SFs group, as determined by ordinary one‐way ANOVA followed by Dunnett's multiple comparisons test. (E) Representative Western blotting analysis showing the effects of p53 siRNA knockdown on p53, Bad, Bax, BID, Bak, Puma, Bcl‐xL, Mcl‐1, and cleaved caspase‐3 (C‐Casp‐3) in Tg‐SFs‐treated hUC‐MSCs. GAPDH served as the loading control. (F) Quantitative densitometric analysis of selected proteins shown in panel (E), including p53, Bax, BID, Puma, Mcl‐1, and C‐Casp‐3. Protein levels were normalized to GAPDH and expressed relative to the control group. Data are presented as mean ± SD. ***p < 0.001, ****p < 0.0001 versus the Tg‐SFs group. Statistical analysis was performed using ordinary one‐way ANOVA followed by Dunnett's multiple comparisons test. Representative Western blot images from one of three independent biological experiments are shown. Densitometric quantification was performed using data from all three independent biological experiments. (G) Schematic model illustrating the proposed mechanism of Tg‐SFs‐induced mitochondrial apoptosis in hUC‐MSCs. Tg‐SFs trigger p53 accumulation and phosphorylation, resulting in increased expression of Bax, BID, and Puma, partial suppression of Mcl‐1, mitochondrial depolarisation, and subsequent caspase‐3 activation. Pharmacological inhibition of p53 with PFT‐α or genetic silencing of p53 with siRNA partially attenuates these pro‐apoptotic responses, indicating that p53 signalling contributes substantially, but not exclusively, to Tg‐SFs‐induced mitochondrial apoptosis. Tg‐SFs: Parasite‐derived soluble factors released from live *T. gondii* tachyzoites in the upper chamber of the Transwell co‐culture system.

Pretreatment with the p53 inhibitor pifithrin‐α (PFT‐α, 10 μM) significantly suppressed Tg‐SFs‐induced phosphorylation of p53 at Ser15 and Ser392. Pharmacological inhibition of p53 attenuated the Tg‐SFs‐induced upregulation of the pro‐apoptotic proteins Bax, BID, and Puma. In contrast, no obvious changes were observed in the expression patterns of Bad and Bak following PFT‐α pretreatment. Likewise, the reduced expression of Bcl‐xL and Mcl‐1 appeared to be only partially restored by PFT‐α treatment (Figure 5A,B). Consistent with these findings, Tg‐SFs markedly increased cleaved caspase‐3 expression, and this effect was significantly attenuated by PFT‐α pretreatment, indicating that p53 contributes to caspase activation downstream of Tg‐SFs exposure.

To further evaluate mitochondrial function, mitochondrial membrane potential (Δψ*m*) was assessed by JC‐1 staining. Tg‐SFs exposure induced pronounced mitochondrial depolarisation, as evidenced by a marked reduction in red JC‐1 aggregates and accumulation of green JC‐1 monomers (Figure 5C). Quantitative analysis demonstrated a significant decrease in the JC‐1 red/green fluorescence ratio following Tg‐SFs exposure, whereas PFT‐α pretreatment partially restored Δψ*m* and increased the red/green fluorescence ratio compared with the Tg‐SFs group (Figure 5D).

To genetically validate the involvement of p53, hUC‐MSCs were transfected with p53 siRNA prior to Tg‐SFs exposure. Western blotting confirmed efficient suppression of p53 expression following siRNA transfection (Figure 5E,F). Importantly, p53 knockdown attenuated the Tg‐SFs‐induced upregulation of Bax, BID, Puma, and cleaved caspase‐3, while partially restoring Mcl‐1 expression. In contrast, no obvious changes were observed in the expression patterns of Bad and Bak following p53 knockdown (Figure 5E,F). These findings provide genetic evidence that p53 contributes to the mitochondrial apoptotic response induced by Tg‐SFs.

Collectively, these results indicate that parasite‐derived soluble factors released from live *T. gondii* tachyzoites induce mitochondrial dysfunction and apoptosis in hUC‐MSCs through a mechanism that is partially dependent on p53 signalling. Pharmacological inhibition or siRNA‐mediated knockdown of p53 attenuated mitochondrial depolarisation, reduced the expression of key pro‐apoptotic Bcl‐2 family proteins, and suppressed caspase‐3 activation, although some apoptotic alterations persisted, suggesting that additional p53‐independent pathways may also contribute to Tg‐SFs‐induced mitochondrial apoptosis. Taken together, these findings indicate that p53 signalling contributes substantially, but not exclusively, to Tg‐SFs‐induced mitochondrial apoptosis in hUC‐MSCs.

## 4 Discussion

In this study, we demonstrate that *T. gondii*‐derived soluble factors (Tg‐SFs), even in the absence of direct parasite invasion, are sufficient to induce cytotoxicity and mitochondrial apoptosis in hUC‐MSCs. Exposure to Tg‐SFs resulted in cytoskeletal remodelling, pronounced accumulation of cells in the G2/M phase, and activation of mitochondrial apoptosis signalling that was partially mediated by p53. These findings extend current understanding of host–parasite interactions by suggesting that parasite‐derived soluble factors released from live extracellular *T. gondii* tachyzoites can compromise hUC‐MSC homeostasis independently of direct intracellular parasitism.

A defining feature of *T. gondii* pathogenesis is its ability to remodel host‐cell architecture to facilitate invasion, intracellular replication, and parasite egress. Previous studies have demonstrated that *T. gondii* infection profoundly dysregulates host cell‐cycle progression and alters cytoskeletal organisation, including microtubule networks and actin dynamics [6, 7]. Consistent with these infection‐based observations, our data show that Tg‐SFs exposure caused marked disruption of F‐actin organisation and α‐tubulin networks in hUC‐MSCs. These findings suggest that soluble parasite‐derived components released in the Transwell co‐culture system can destabilize cytoskeletal integrity even without direct parasite invasion. This interpretation is also supported by evidence that *T. gondii* excretory/secretory antigens can disrupt intracellular junction proteins and cytoskeletal organisation in epithelial monolayers, underscoring the intrinsic cytotoxic potential of parasite‐derived soluble products [21].

MSCs rely heavily on an intact and dynamic cytoskeleton for proliferation, migration, mechanotransduction, and lineage commitment [22]. Cytoskeletal integrity is closely coupled to MSC self‐renewal capacity and functional plasticity [23]. Therefore, Tg‐SFs‐induced cytoskeletal remodelling may have important consequences for MSC survival and regenerative function. Beyond direct infection, *T. gondii* can influence host‐cell behaviour through parasite‐derived soluble mediators, including EVs, which carry parasite proteins and regulatory RNAs capable of modulating host immune and stress‐response pathways [24]. These observations provide a plausible mechanistic basis for the cytotoxic and stress‐related responses observed following Tg‐SFs exposure. However, because the present study did not include non‐stem‐cell controls, we cannot determine whether the observed phenotype is specific to hUC‐MSCs or represents a broader cytotoxic response shared by other cell types. Accordingly, our conclusions are restricted to hUC‐MSCs, and future comparative studies using non‐stem‐cell controls and MSCs from other tissue sources will be required to clarify cell‐type specificity.

Cell‐cycle dysregulation is a recognised consequence of *T. gondii* infection, traditionally attributed to intracellular replication and effector translocation [7, 25]. In the present study, Tg‐SFs exposure induced marked G2/M phase accumulation in hUC‐MSCs, accompanied by downregulation of Cyclin A2, Cyclin B1, and Cyclin E2, upregulation of p21 (Waf1/Cip1), and reduced phosphorylation of CDK1 at Thr161. Because Thr161 phosphorylation is required for full CDK1 activation, this finding is consistent with impaired CDK1 activation associated with G2/M phase accumulation. These findings indicate that parasite‐derived soluble factors are capable of perturbing cell‐cycle regulation independently of direct parasite invasion. Altered microtubule organisation may further contribute to altered G2/M progression and apoptotic susceptibility, although additional experiments are required to define the precise upstream events linking cytoskeletal remodelling to cell‐cycle accumulation.

Mechanistically, our data support a contributory role of p53 signalling in Tg‐SFs‐induced mitochondrial apoptosis. Tg‐SFs exposure resulted in accumulation of total p53 protein and enhanced p53 phosphorylation at Ser15 and Ser392, whereas additional phosphorylation sites were either undetectable or showed no obvious change (Figure S2). These results suggest selective activation of p53 signalling in response to Tg‐SFs‐induced cellular stress. Downstream of p53 activation, Tg‐SFs promoted the expression of several pro‐apoptotic Bcl‐2 family proteins, including Bax, BID, and Puma, and induced mitochondrial depolarization, cleaved caspase‐3 activation, and PARP cleavage. Pharmacological inhibition of p53 with PFT‐α and siRNA‐mediated p53 knockdown both attenuated key apoptotic responses, supporting the involvement of p53 in this process.

Importantly, the protective effects of PFT‐α and p53 knockdown were partial rather than complete. PFT‐α reduced Tg‐SFs‐induced p53 phosphorylation, pro‐apoptotic protein expression, mitochondrial depolarisation, and caspase‐3 activation, but did not fully restore all apoptotic markers. Similarly, p53 knockdown attenuated Bax, BID, Puma, and cleaved caspase‐3 induction, while only partially restoring Mcl‐1 expression. These findings indicate that p53 contributes substantially to Tg‐SFs‐induced mitochondrial apoptosis but is unlikely to be the sole mediator. Additional p53‐independent mechanisms, potentially involving proteasomal regulation, ER stress, oxidative stress, or other stress‐responsive signalling pathways, may also participate in the apoptotic response [26, 27]. Based on these findings, we propose a working model in which Tg‐SFs activate p53 signalling, resulting in upregulation of the pro‐apoptotic proteins Bax, BID, and Puma, partial suppression of the anti‐apoptotic protein Mcl‐1, mitochondrial depolarisation, and subsequent activation of cleaved caspase‐3, ultimately leading to apoptotic cell death in hUC‐MSCs (Figure 5G).

From a translational perspective, these findings raise important considerations for stem‐cell‐based therapies. Cytoskeletal remodelling, G2/M phase accumulation, and mitochondrial apoptosis may impair MSC proliferation, migration, survival, and long‐term functional persistence, thereby potentially limiting tissue repair efficacy. Given the high global prevalence of latent toxoplasmosis [1], parasite‐derived soluble factors may represent previously underappreciated modifiers of MSC behaviour in parasite‐exposed populations [12, 27, 28]. However, because our data were obtained in an in vitro Transwell co‐culture model, the clinical relevance of these findings remains to be validated in more physiologically relevant systems.

This study used biochemically characterised ESP preparations and a Transwell‐based Tg‐SFs exposure system to investigate the biological effects of parasite‐derived soluble factors. Although our in vitro system cannot fully recapitulate the complexity of stem‐cell niches in vivo, it demonstrates that Tg‐SFs profoundly disrupt MSC homeostasis. Future studies employing component‐resolved analyses of parasite‐derived soluble factors and in vivo or organoid‐based models will be required to elucidate upstream signalling pathways linking Tg‐SFs exposure to p53 activation.

In conclusion, this study reveals a previously unrecognised invasion‐independent mechanism by which *T. gondii*‐derived soluble factors compromise hUC‐MSC survival and homeostasis. Tg‐SFs induce cytoskeletal remodelling, G2/M phase accumulation, and mitochondrial apoptosis that is partially mediated by p53 signalling and accompanied by coordinated modulation of Bax, BID, Puma, Mcl‐1, and downstream cleaved caspase‐3 activation. These findings provide new insight into how parasite‐derived soluble factors may affect MSC biology and highlight the need to consider infectious or parasite‐derived factors in the context of regenerative medicine.

## Author Contributions

**Tingyu Chen:** writing – original draft, investigation, methodology, data curation, formal analysis, visualization. **Jie Liu:** project administration, software. **Jinjing Huang:** investigation, methodology. **Jiaqi Chu:** conceptualization, supervision, investigation, resources, writing – review and editing. **Wei Zhou:** conceptualization, data curation, writing – original draft, investigation, methodology, formal analysis, validation. **Juanhua Quan:** writing – review and editing, conceptualization, supervision, funding acquisition, investigation. **Xinyi Lai:** validation, software, investigation. **Ziping Yang:** methodology, investigation, software.

## Funding

This study was supported by the High‐level Talents Scientific Research Start‐up Funds of the Affiliated Hospital of Guangdong Medical University (Grant No. GCC2022010) and the National Natural Science Foundation of China (Grant No. 82211540401).

## Ethics Statement

This study was approved by the Ethics Committee of the Affiliated Hospital of Guangdong Medical University, and written informed consent was obtained from all donors.

## Conflicts of Interest

The authors declare no conflicts of interest.

## Supporting information


**Figure S1:** Flow cytometric gating strategy for Annexin V‐FITC/PI apoptosis analysis of hUC‐MSCs. Cell debris was excluded using FSC‐A versus SSC‐A gating, and doublets were removed using FSC‐H versus FSC‐A gating to obtain a single‐cell population. Apoptosis was subsequently assessed using Annexin V‐FITC/PI staining. Q4 represents viable cells (Annexin V^−^/PI^−^), Q3 represents early apoptotic cells (Annexin V^+^/PI^−^), Q2 represents late apoptotic cells (Annexin V^+^/PI^+^), and Q1 represents necrotic cells (Annexin V^−^/PI^+^). Representative gating plots from the Ctrl and Tg‐SFs groups are shown.
**Figure S2:** Original Western blotting images corresponding to the screening of additional p53 phosphorylation sites in hUC‐MSCs exposed to Tg‐SFs. Tg‐SFs were defined as parasite‐derived soluble factors released from live Toxoplasma gondii tachyzoites placed in the upper chamber of the Transwell co‐culture system (at an upper‐chamber MOI of 50) for 24 h. Groups from left to right: Ctrl and Tg‐SFs. The PageRuler Prestained Protein Ladder was used as the molecular weight marker. No specific bands at the expected molecular weight of approximately 53 kDa were detected for p‐p53 (Ser6), p‐p53 (Ser9), p‐p53 (Ser46), or p‐p53 (Thr81) in either control or Tg‐SFs‐treated hUC‐MSCs. The visible off‐target band in the p‐p53 (Ser9) blot did not correspond to the expected molecular weight and was considered nonspecific. p‐p53 (Ser20) showed no obvious change following Tg‐SFs exposure. The GAPDH band (~37 kDa) was aligned between the 35 and 40 kDa reference bands and served as the loading control.

**Data S1:** Supplementary Methods. Detailed experimental procedures for qRT‐PCR, Western blotting, flow cytometry, statistical analysis, and endotoxin detection.

## Data Availability

Research data are not shared.
