# Supplementary material for: Toxoplasma gondii‐Derived Soluble Factors Induce G2/M Phase Accumulation and p53‐Associated Mitochondrial Apoptosis in Human Umbilical Cord Mesenchymal Stem Cells
Source: J Cell Mol Med. 2026 Aug 16;30(16):e71312. doi: 10.1111/jcmm.71312 (PMC13478567; doi:10.1111/jcmm.71312)
Supplement: Supplementary file 1 [file JCMM-30-e71312-s002.pdf]

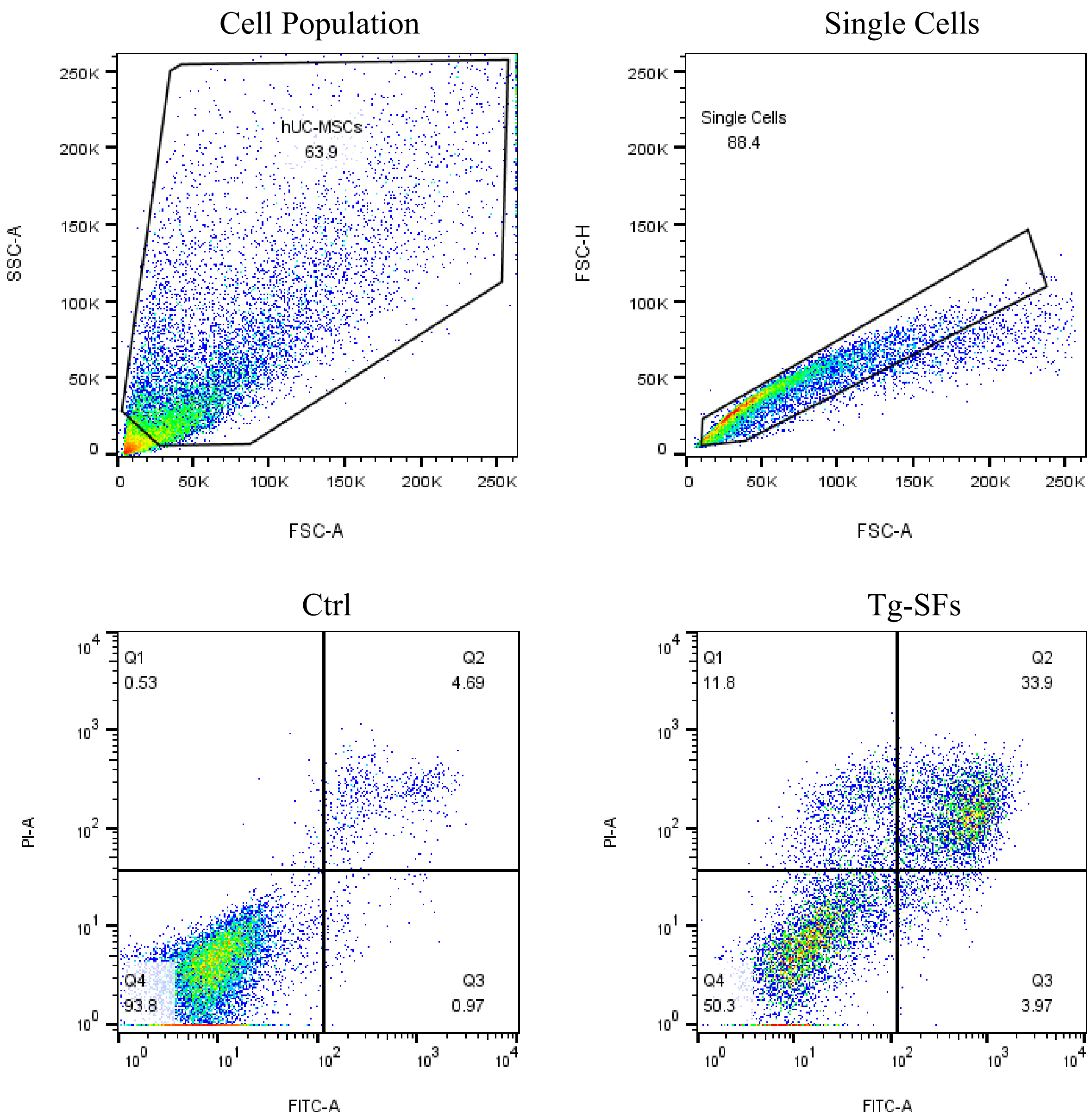

Supplementary Figure S1. Flow cytometric gating strategy for Annexin V-FITC/PI apoptosis analysis of hUC-MSCs. Cell debris was excluded using FSC-A versus SSC-A gating, and doublets were removed using FSC-H versus FSC-A gating to obtain a single-cell population. Apoptosis was subsequently assessed using Annexin V-FITC/PI staining. Q4 represents viable cells (Annexin V^-/PI^-), Q3 represents early apoptotic cells (Annexin V^+/PI^-), Q2 represents late apoptotic cells (Annexin V^+/PI^+), and Q1 represents necrotic cells (Annexin V^-/PI^+). Representative gating plots from the Ctrl and Tg-SFs groups are shown.

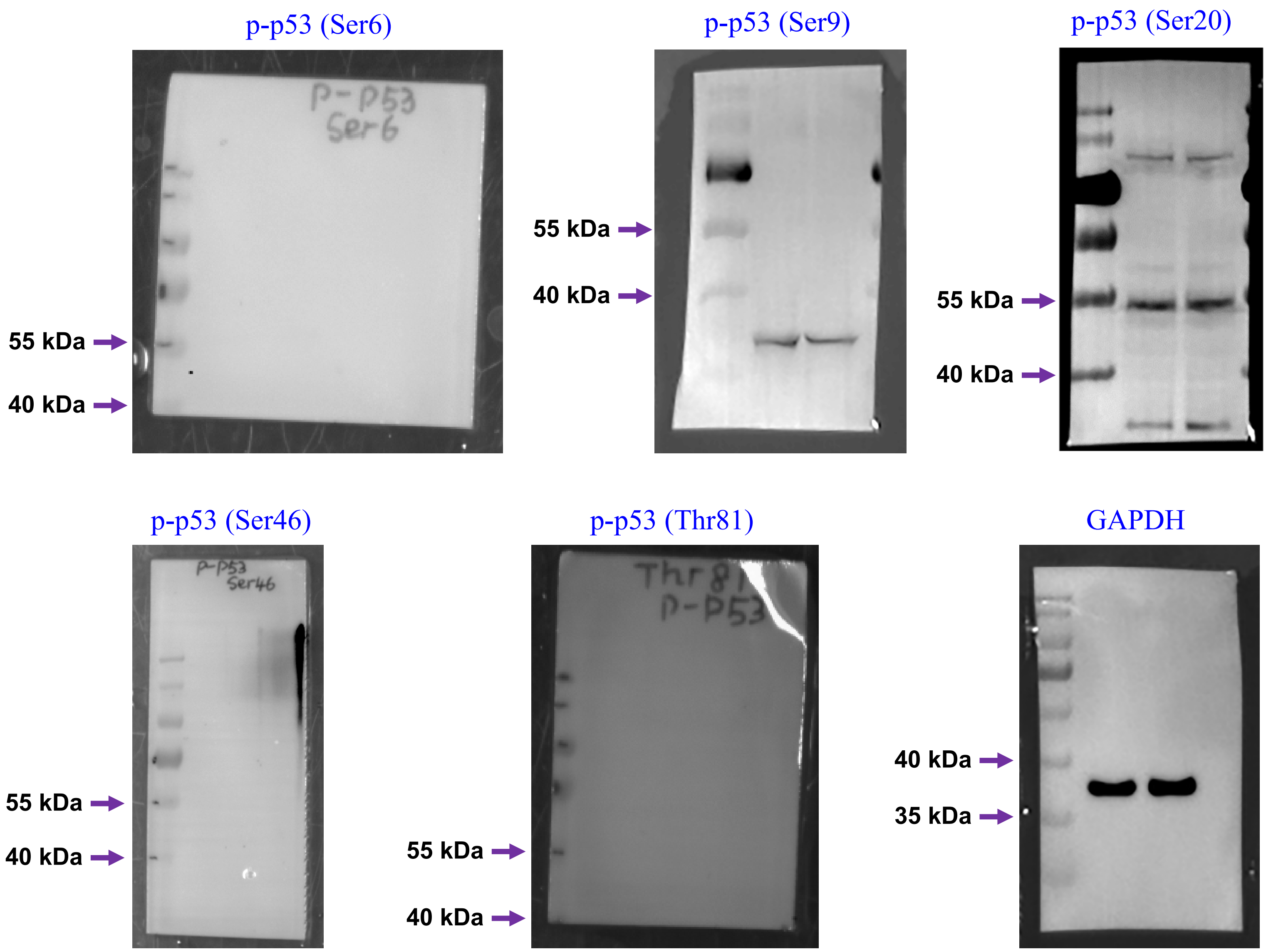

Supplementary Figure S2. Original Western blotting images corresponding to the screening of additional p53 phosphorylation sites in hUC-MSCs exposed to Tg-SFs. Tg-SFs were defined as parasite-derived soluble factors released from live *T. gondii* tachyzoites placed in the upper chamber of the Transwell co-culture system (at an upper-chamber MOI of 50) for 24 h. Groups from left to right: Ctrl and Tg-SFs. The PageRuler Prestained Protein Ladder was used as the molecular weight marker. No specific bands at the expected molecular weight of approximately 53 kDa were detected for p-p53 (Ser6), p-p53 (Ser9), p-p53 (Ser46), or p-p53 (Thr81) in either control or Tg-SFs-treated hUC-MSCs. The visible off-target band in the p-p53 (Ser9) blot did not correspond to the expected molecular weight and was considered nonspecific. p-p53 (Ser20) showed no obvious change following Tg-SFs exposure. The GAPDH band (~37 kDa) was aligned between the 35 kDa and 40 kDa reference bands and served as the loading control.
