# Supplementary material for: Toxoplasma gondii‐Derived Soluble Factors Induce G2/M Phase Accumulation and p53‐Associated Mitochondrial Apoptosis in Human Umbilical Cord Mesenchymal Stem Cells
Source: J Cell Mol Med. 2026 Aug 16;30(16):e71312. doi: 10.1111/jcmm.71312 (PMC13478567; doi:10.1111/jcmm.71312)
Supplement: Supplementary file 2 [file JCMM-30-e71312-s001.docx]

**Supplementary Methods**

**qRT-PCR conditions**

Total RNA was extracted using TRIzol Reagent (Invitrogen, Carlsbad, CA) according to the manufacturer’s instructions. The purified RNA was reverse-transcribed into cDNA using M-MLV reverse transcriptase (Invitrogen Life Technologies). Quantitative real-time PCR (qRT-PCR) was performed using Power SYBR® Green PCR Master Mix (Applied Biosystems, Foster City, CA) on an ABI 7500 Fast Real-Time PCR System (Applied Biosystems, Carlsbad, CA). Primer sequences used in this study are listed in Table 1. The amplification protocol consisted of an initial denaturation at 95°C for 10 min, followed by 40 cycles of 95°C for 15 s and 60°C for 60 s. Relative gene expression levels were normalized to hypoxanthine phosphoribosyltransferase 1 (HPRT1) and calculated using the 2^−ΔΔCt^ method. Three independent biological experiments were performed unless otherwise indicated, and each sample was analyzed in triplicate.

**Western blotting details**

Human umbilical cord-derived mesenchymal stem cells (hUC-MSCs) were washed with phosphate-buffered saline (PBS), and total cellular proteins were extracted using radioimmunoprecipitation assay (RIPA) buffer (Thermo Scientific, Cat. No. 89900) supplemented with a protease inhibitor cocktail (Roche, Cat. No. 5892791001). Cell lysates were incubated on ice for 30 min and centrifuged at 14,000 ×g for 15 min at 4°C.

Protein concentrations were determined using a BCA Protein Assay Kit (Beyotime, Cat. No. P0010) according to the manufacturer’s instructions. Equal amounts of protein (30 μg per lane) from Lys and ESP preparations were separated by sodium dodecyl sulfate-polyacrylamide gel electrophoresis (SDS-PAGE) and transferred onto polyvinylidene difluoride (PVDF) membranes (Millipore, Cat. Nos. ISEQ00010 and IEVH00005). A Thermo Scientific PageRuler Prestained Protein Ladder (Cat. No. 26617) was loaded as the molecular weight marker. Membranes were blocked with 5% non-fat milk in Tris-buffered saline containing 0.1% Tween-20 (TBST) and incubated with primary antibodies overnight at 4°C.

The primary antibodies used in this study included p-CDK1 (Tyr15) (MCE, HY-P80796), p-CDK1 (Thr161) (Abcam, ab138389), CDK1 (MCE, HY-P80611), Cell Cycle Regulation Antibody Sampler Kit II (CST, #9870), PARP (CST, #9532), Cleaved Caspase-3 (CST, #9664), Phospho-p53 Antibody Sampler Kit (CST, #65390; recognizing p53 phosphorylated at Ser6, Ser9, Ser15, Thr81, Ser20, Thr18, Ser37, Ser46, and Ser392), Pro-Apoptosis Bcl-2 Family Antibody Sampler Kit (CST, #9942), Pro-Survival Bcl-2 Family Antibody Sampler Kit (CST, #9941), TP3 anti-*Toxoplasma gondii antibody* (Santa Cruz Biotechnology, sc-52255), α-Tubulin (Santa Cruz Biotechnology, sc-32293), and GAPDH (CST, #2118). Unless otherwise specified by the manufacturer, primary antibodies were used at a dilution of 1:1000.

After washing three times with TBST, membranes were incubated with horseradish peroxidase (HRP)-conjugated anti-rabbit or anti-mouse secondary antibodies (Cell Signaling Technology, #7074 and #7076, respectively) at a dilution of 1:5000 for 2 h at room temperature. Protein signals were visualized using Immobilon Western Chemiluminescent HRP Substrate (Millipore, Cat. No. WBKLS0500) and captured with a Tanon 5200 chemiluminescence imaging system (Tanon Science & Technology Co., Ltd., Shanghai, China).

Band intensities were quantified using ImageJ software (National Institutes of Health, Bethesda, MD, USA). Relative protein expression levels were normalized to the corresponding loading control (GAPDH or α-Tubulin, as appropriate) and expressed as fold changes relative to the control group. For phosphorylated proteins, densitometric values were normalized to the corresponding total protein whenever available; otherwise, normalization was performed using the appropriate loading control. The 17/19 kDa cleaved caspase-3 doublet was quantified as a single combined signal for densitometric analysis.

**Flow cytometry staining and gating strategy**

Apoptosis detection by FITC Annexin V/PI

Cells were treated as described in the corresponding experiments and harvested after 24 h for apoptosis analysis. Both floating and adherent cells were collected, washed twice with cold PBS, and resuspended in 500 μL binding buffer. The cell suspension was incubated with 5 μL Annexin V-FITC and 5 μL PI for 15 min at room temperature in the dark. Stained cells were analyzed using a FACSCanto II flow cytometer. Cell debris was excluded using FSC-A versus SSC-A gating, and doublets were excluded using FSC-H versus FSC-A gating. A minimum of 10,000 events were acquired for each sample. Early apoptotic cells were defined as Annexin V-positive/PI-negative, whereas late apoptotic cells were defined as Annexin V-positive/PI-positive. Flow cytometry data were analyzed using FlowJo software (BD Biosciences, Ashland, OR, USA). The gating strategy used for apoptosis analysis is shown in Supplementary Figure S1.

**Endotoxin detection**

Endotoxin levels in experimental samples were measured using a highly sensitive endotoxin assay kit (Beyotime, Cat. No. C02775S) according to the manufacturer's instructions. Briefly, endotoxin-free water was used as the negative control, and a 0.1 EU/mL endotoxin standard served as the positive control. The tested samples included ARPE-19 culture medium alone, ARPE-19 cell culture supernatant, supernatant from ARPE-19 cultures infected with *T. gondii* tachyzoites (MOI 5), isolated *T. gondii* ESP preparation (30 μg protein), hUC-MSC culture supernatant, and Transwell co-culture supernatants collected from hUC-MSCs exposed to live *T. gondii* tachyzoites at upper-chamber MOIs of 0, 10, 20, 50, 80, and 100. All tested samples exhibited endotoxin levels below the assay detection threshold, indicating that the biological effects reported in this study were unlikely to be attributable to endotoxin contamination.

**Statistical analysis**

Unless otherwise indicated, all experiments were performed using three independent biological replicates, and data are presented as mean ± standard deviation (SD). Statistical analyses were performed using GraphPad Prism 10.0 software (GraphPad Software, San Diego, CA, USA).

For comparisons between two groups, an unpaired two-tailed Student’s t-test was used. For comparisons among three or more groups, one-way analysis of variance (ANOVA) followed by Dunnett’s multiple-comparisons test was applied when all treatment groups were compared with a single control group. When variance homogeneity assumptions were not satisfied, as determined by Bartlett’s test, Brown-Forsythe and Welch ANOVA followed by Dunnett’s T3 multiple-comparisons test was used.

For fluorescence image analysis, at least 20 randomly selected cells were quantified per experimental group using ImageJ software (National Institutes of Health, Bethesda, MD, USA), and fluorescence intensity was normalized on a per-cell basis (integrated density/area). Differences were considered statistically significant at *P* < 0.05. Statistical significance is indicated as follows: ^*^*P* < 0.05, ^**^*P* < 0.01, ^***^*P* < 0.001, and ^****^*P* < 0.0001.
